# Computational reconstruction reveals a candidate magnetic biocompass to be likely irrelevant for magnetoreception

**DOI:** 10.1038/s41598-017-13258-7

**Published:** 2017-10-24

**Authors:** Ida Friis, Emil Sjulstok, Ilia A. Solov’yov

**Affiliations:** Department of Physics, Chemistry and Pharmacy, University of Southern Denmark, DK-5230 Odense M, Denmark

## Abstract

Birds use the magnetic field of the Earth to navigate during their annual migratory travel. The possible mechanism to explain the biophysics of this compass sense involves electron transfers within the photoreceptive protein cryptochrome. The magnetoreceptive functioning of cryptochromes is supposedly facilitated through an iron rich polymer complex which couples to multiple cryptochromes. The present investigation aims to independently reconstruct this complex and describe its interaction with *Drosophila melanogaster* cryptochromes. The polymer complex consists of ISCA1 protein monomers with internally bound iron sulphur clusters and simultaneously binds ten cryptochromes. Through molecular dynamics we have analysed the stability of the ISCA1-cryptochrome complex and characterized the interaction at the binding sites between individual cryptochrome and ISCA1. It is found that the cryptochrome binding to the ISCA1 polymer is not uniform and that the binding affinity depends on its placement along the ISCA1 polymer. This finding supports the claim that the individual ISCA1 monomer acts as possible intracellular interaction partner of cryptochrome, but the proposed existence of an elongated ISCA1 polymer with multiple attached cryptochromes appears to be questionable.

## Introduction

Since the discovery of migration in animals, it has baffled scientists how certain migratory animals find their way around the globe without getting lost^1–3^. It is well-documented experimentally that, among other cues, migratory birds use magnetic fields^2,4–8^ for their navigation. A popular explanation for this phenomenon is provided through the so-called radical pair mechanism^1,2,9–15^, which links the magnetic sense to a quantum mechanical effect where an entangled pair of unpaired electron spins can interact with the Earth’s magnetic field and thereby eventually influence avian behaviour. Theoretically a radical pair could be described though the Hamilton operator that includes interactions between unpaired electrons and nuclei, the so-called hyperfine interactions, as well as the Zeeman interaction between the magnetic field and the electrons. Due to those interactions, the magnetic field influences the total spin character of the radical pair, continuously altering it between the triplet and the singlet states. The singlet and triplet states of a radical pair in turn act chemically different, giving raise to the so-called singlet and triplet products^1,2,9–15^. The ratio between these products could be governed by the magnitude and direction of the magnetic field and it is, therefore, argued^9–14^ that such a reaction could form the basis of the chemical compass of migratory birds.

The radical pair reaction could in principle occur anywhere inside a bird, and currently the most promising host molecule is the blue light photoreceptor protein called cryptochrome (Cry)^1,2,9,14–21^. Cryptochrome is a common protein found in many organisms, and particularly in birds’ eyes^22–24^. It has the remarkable property to become biologically active through creation of transient radical pairs involving the flavin adenine dinucleotide (FAD) cofactor and three tryptophan residues through a series of electron transfer reactions^9,15,19,25–27^. The crystal structure of Cry itself is known for at least some species such as *Arabidopsis thaliana* (plant)^28^, *Drosophila melanogaster* (insect)^29^ and *Mus musculus* (mammal)^30^. However it is still not crystallized for birds and only advanced homology models are available.

Even though the structures of some cryptochromes are documented, the protein cannot act in isolation, but will interact with other proteins inside the cell^2,14^. Such an interaction partner could potentially have great importance for the magnetoreception functioning of cryptochrome as it could be the source of the necessary properties of a magnetic field sensitive radical pair, such as for example large separation distance^31^ or large spin coherence and relaxation times^32–34^. Unfortunately no crystallized binding partners that could potentially boost cryptochrome magnetoreception are known yet.

**Figure 1:**
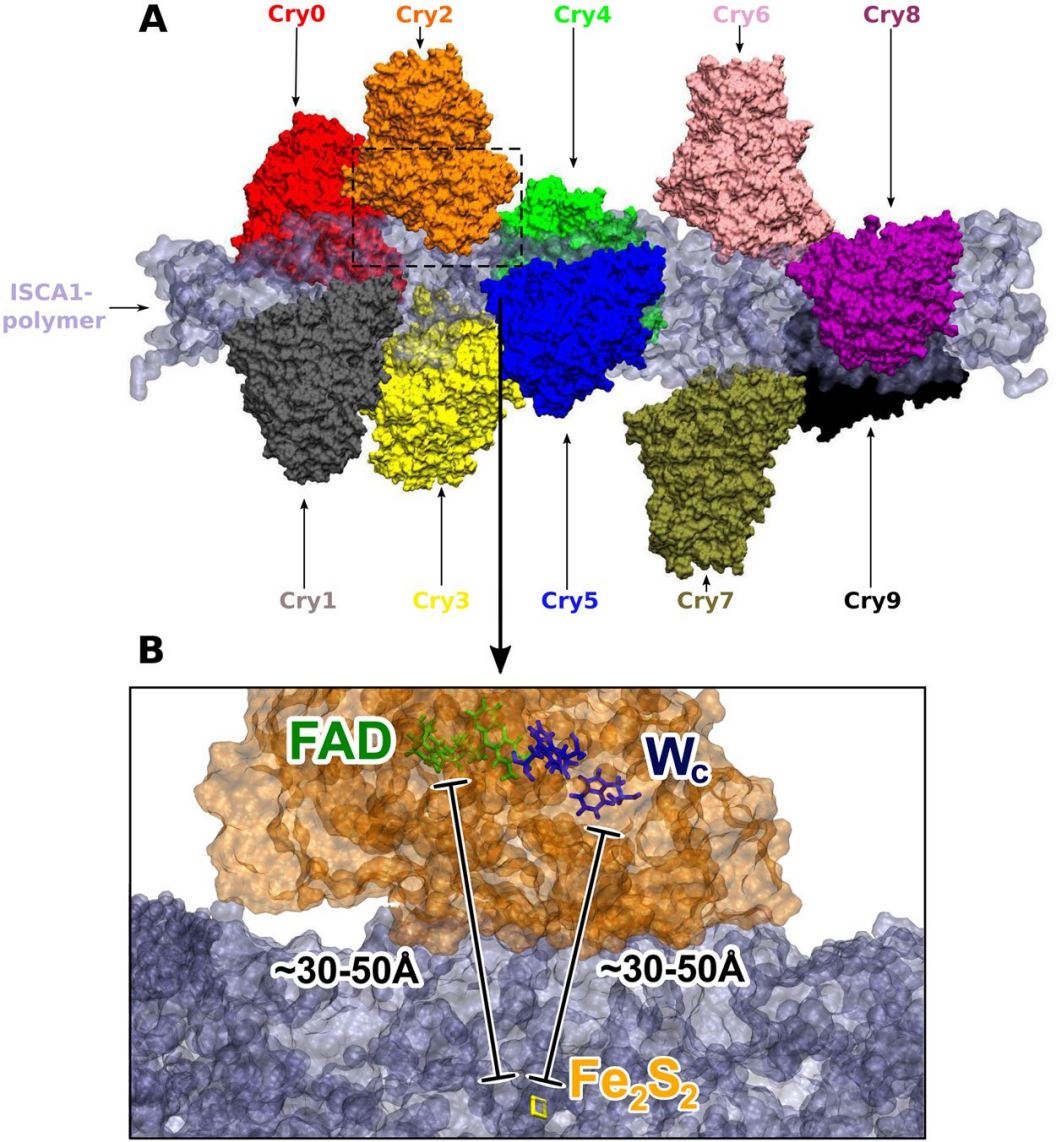
Molecular structure of the ISCA1-Cry complex. (**A**) Ten different cryptochromes are shown attached to the ISCA1-polymer. The cryptochromes here are assigned a number and a colour, which are later used for analysis purposes. The surface of the ISCA1-polymer is shown in pale blue. (**B**) A zoom in on Cry2 featuring the FAD cofactor, the tryptophan triad, and the closest Fe_2_S_2_ cluster from a nearby ISCA1 monomer. Here the contact distance between the FAD cofactor and the nearest Fe_2_S_2_-cluster as well as the contact distance between the terminal tryptophan, W_C_, and the Fe_2_S_2_-cluster is indicated.

A recent study^35^ has proposed the existence of a rod-like protein polymer consisting of multiple iron sulphur (Fe_2_S_2_) assembly proteins called ISCA1. This polymer was presented as a possible binding partner to several cryptochromes, as well as having intrinsic magnetic properties that putatively enhances migratory birds’ sensitivity to changes in the Earth’s magnetic field. Figure 1 illustrates the spatial arrangement of the ISCA1-complex binding 10 cryptochromes, as originally suggested^35^. The figure also indicates the FAD moiety, and tryptophan triad in an individual cryptochrome, being crucial for its functioning^19,20^. The role of the ISCA1-Cry complex in migration was claimed^35^ to be that of a biological compass needle, arising due to the presence of iron atoms within the ISCA1 polymer, giving it bar magnet-like properties. These claims, however, raised a strong critique^36–39^, as the original study^35^ left a number of unanswered questions. Therefore it is widely accepted now^36–38^ that the role of ISCA1-Cry complex in magnetoreception and the possible interaction between ISCA1 and cryptochromes requires independent verification.

In the present investigation we consider the computational aspects of the original paper^35^ and study the ISCA1-Cry complex though computational modelling. The complex is constructed largely following the protocol of Qin *et al*.^35^, and the validity of the model is discussed though analysis of the ISCA1-Cry complex stability and dynamics. The binding between cryptochrome and ISCA1 is investigated and characterized to conclude if the interaction is favourable. The study reveals some major differences to the original ISCA1-Cry structure that may shed light on the realistic feasibility of the ISCA1-Cry complex and suggests that it is unlikely to be relevant for radical pair based magnetoreception.

## Results

### Stability of the structure

**Figure 2:**
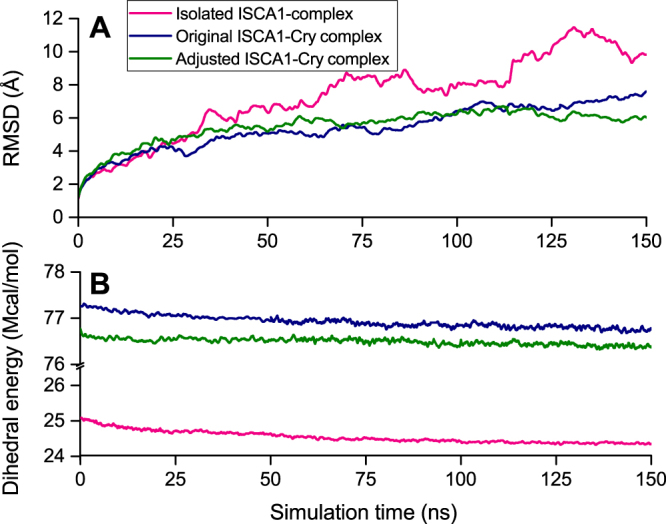
Stability of the ISCA1-Cry complex. (**A**) The time evolution of the root mean square displacement (RMSD) computed for the original ISCA1-Cry (blue) and the adjusted ISCA1-Cry (green) complexes. Both dependencies are similar, whereas the isolated ISCA1-complex (red) has a noticeably higher RMSD. (**B**) The time evolution of the dihedral energies, Eq. (1), computed for the three different systems studied. The original ISCA1-Cry (blue) and the adjusted ISCA1-Cry (green) are similar whereas the ISCA1-complex (red) has a much lower energy.

**Figure 3:**
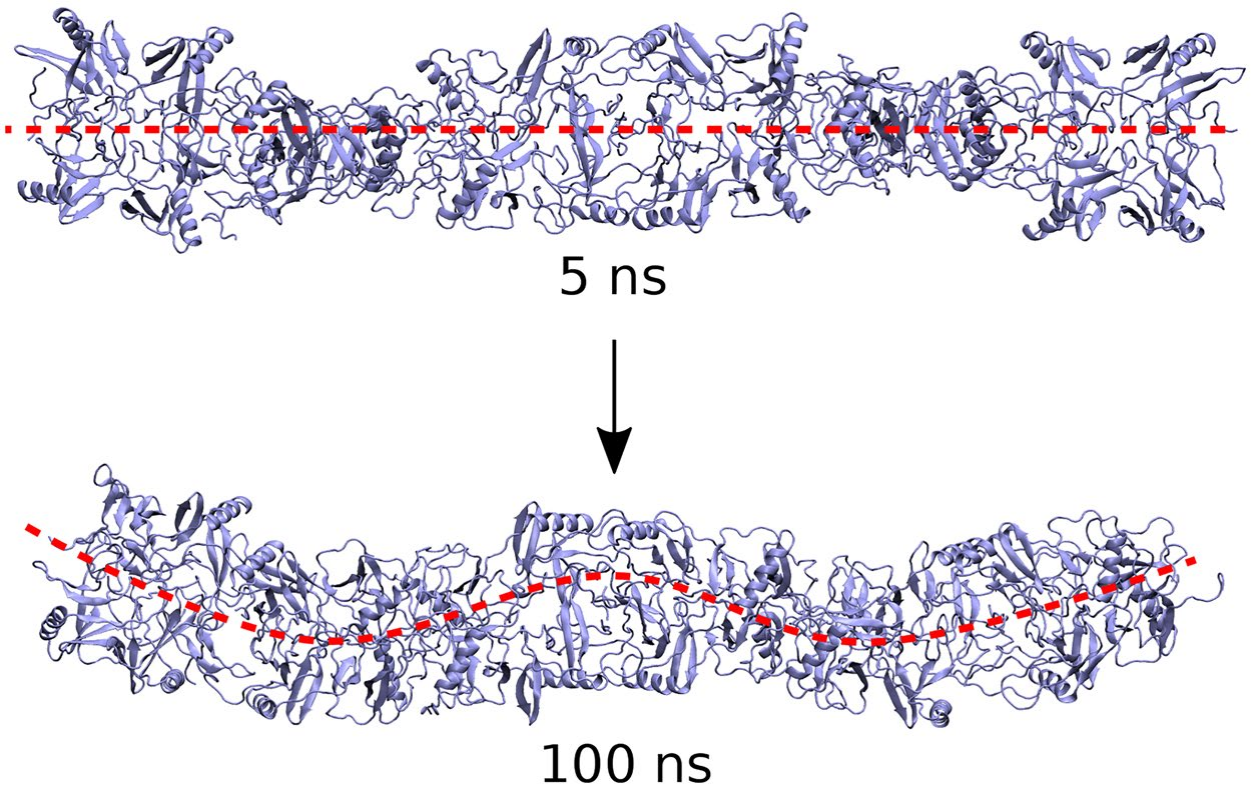
The large scale motion of the ISCA1-polymer. The secondary structure of the ISCA1 polymer is shown in light blue. After an 100 ns simulation, the ISCA1-polymer has transformed into an S-shape, as indicated by the dashed red line.

Stability of a macromolecular structure can be described through the time evolution of the root mean square displacement (RMSD) of its individual atoms. The results for the root mean square displacement for the ISCA1-complex are shown in Fig. 2A (red line), which features a rather large RMSD value evolving to about 10 Å after the 150 ns simulation, and does not show any pronounced tendency to stability at the end of the simulation. The continuous increase of RMSD for the ISCA1-polymer can be ascribed to the geometry of the polymer, as its elongated shape allows for long term twisting or bending motions. Indeed, already after 100 ns simulations the ISCA1-polymer was found to bend into a distinct S-shape, as featured in Fig. 3. This does not necessarily mean that the structure is unstable, but such a large scale motion will result in an increased RMSD value.

Figure 2A reveals that the computed RMSD of the ISCA1-Cry structure (blue line) is notably lower than for the ISCA1-polymer itself, as the RMSD of the ISCA1-Cry complex converges to ~6 Å compared to the ~10 Å value of the isolated ISCA1 polymer. This indicates that adding the cryptochromes to the elongated structure of the ISCA1-polymer suppresses the bending motion of the polymer. Furthermore the lower RMSD values indicates that the attachment of cryptochromes to the ISCA1 polymer is not an unfavourable process.

A better characteristic to conclude about structural stability is the internal energy of the ISCA1 and ISCA1-Cry structures. Once the structure reaches equilibrium the energy should become minimal, providing a better estimator for the stability of the ISCA1 polymer. The total energy of the system, however, is not a good measure here, as the motion of the water molecules and salt ions in the solution will contribute significantly to its value making it difficult to distinguish between the contribution from the protein complex and the contribution from the solvent. To extract the energy associated solely with the conformational dynamics of the ISCA1 polymer, the dihedral energy is calculated, which within the CHARMM force field is parametrized as^40–42^:

1 $${U}_{{\rm{dihedral}}}=\sum _{i\in {\rm{dihedrals}}}{k}_{i}[1+\,\cos ({n}_{i}{\theta }_{i}-{\delta }_{i})],$$

where *θ*_*i*_ is a dihedral angle defined by a quadruple of atoms, *n*_*i*_ is the multiplicity of the corresponding dihedral interaction, *δ*_*i*_ is the associated phase and *k*_*i*_ is the interaction stiffness parameter.

The red line in Fig. 2B shows that dihedral energy for the ISCA1 polymer decreases slowly during the simulation, indicating that the structure is tending towards an equilibrium, however, it also reveals that the final structure is not stabilized entirely after the performed 150 ns simulation. Figure 2B shows that the dihedral energy of the ISCA1-Cry complex is larger than the dihedral energy of the ISCA1-polymer, which is simply due to the larger amount of atoms and dihedral interactions in the structure. The dihedral energy of the ISCA1-Cry complex shows a descending behaviour that indicates that the complex is also tending towards an equilibrium. Neither the dihedral energy or the RMSD however, yields insight of the specific binding sites of the cryptochrome to the ISCA1-polymer.

### Possible electron transfer to cryptochrome from the ISCA1

Qin *et al*.^35^ suggested a possible electron transfer between the iron atoms of in the Fe_2_S_2_ cluster in the ISCA1 polymer and cryptochrome. In this respect, the FAD cofactor and the tryptophan triad are particularly interesting as these components constitute the radical pair in isolated cryptochrome^15,31,33^. In the triad the tryptophan denoted W_C_ is especially interesting as it acts as an electron donor on cryptochrome periphery.

As electron transfers between the cyptochrome and its interaction partner are possibly crucial^14,43^ for the operation of the molecular biocompass in migratory birds, the likelihood of such an event to happen is investigated by measuring the distance between the FAD cofactor and the Fe_2_S_2_-cluster, as well as between W_C_ and the Fe_2_S_2_ cluster. According to the Marcus theory of electron transfer^44^, the rate constant of an electron transfer reaction is proportional to the electronic coupling between the orbitals of the donor and acceptor state, *H*_*DA*_; where

2 $${|{H}_{{\rm{DA}}}|}^{2}\sim {V}_{0}^{2}\exp (-\beta R).$$

Here *V*_0_ is the coupling coefficient between the acceptor and the donor states, *β* is the characteristic decay length parameter and *R* is the distance between the acceptor and the donor. Due to the exponential function, the rate is heavily dependant on the distance between the donor and the acceptor, which can be estimated as the edge-to-edge distance between the sites.

**Figure 4:**
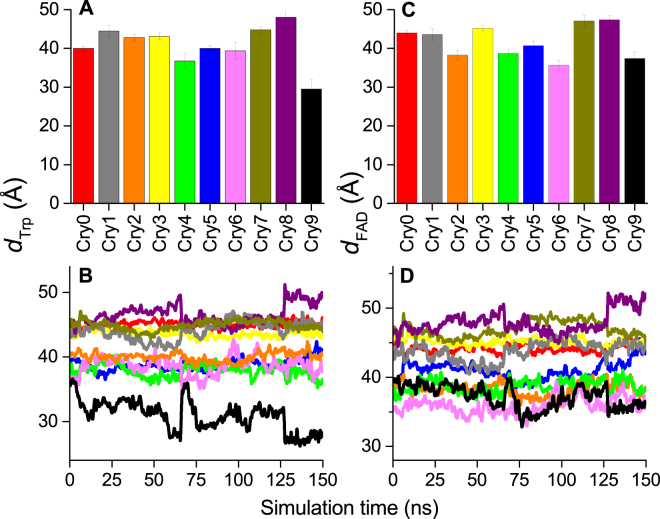
The distance between the radicals in cryptochrome and the nearest Fe_2_S_2_-cluster. The computed distances between the termial tryptophan W_C_ in each cryptochrome and the nearest Fe_2_S_2_-cluster, denoted *d*_Trp_, and the distance between the FAD cofactor in each cryptochrome and the nearest Fe_2_S_2_-cluster, denoted *d*_FAD_. (**A**) The mean value of the contact distance, *d*_Trp_, and the corresponding standard deviation computed from the performed MD simulation. (**B**) The time evolution of *d*_Trp_ in each individual cryptochrome is shown, colour coded following the notation introduced in Fig. 1A. (**C**) The mean value of the contact distance, *d*_FAD_, computed from the performed MD simulation. (**D**) The time evolution of *d*_FAD_ in each individual cryptochrome is shown, colour coded following the notation introduced in Fig. 1A.

The edge-to-edge distance between FAD and the nearest Fe_2_S_2_-cluster as well as the edge-to-edge distance between W_C_ and the nearest Fe_2_S_2_-cluster was measured throughout the simulation and turned out to be between 30–50 Å during the entire simulation for all the 10 studied cryptochromes, as seen in Fig. 4. This distance is significantly larger than what was implied in the suggestion of Qin *et al*.^35^, and has emerged after the dynamical study performed here. Such a distance is too large to allow any electron transfer at biologically reasonable time-scale of nanoseconds and beyond^31,43,45^, and, according to Eq. (2), it would lead to a diminishing electron transfer rate constant which makes it problematic for the ISCA1-polymer to play a significant role in the radical pair mechanism of avian magnetoreception.

### Interaction energy between ISCA1 and Cry

**Figure 5:**
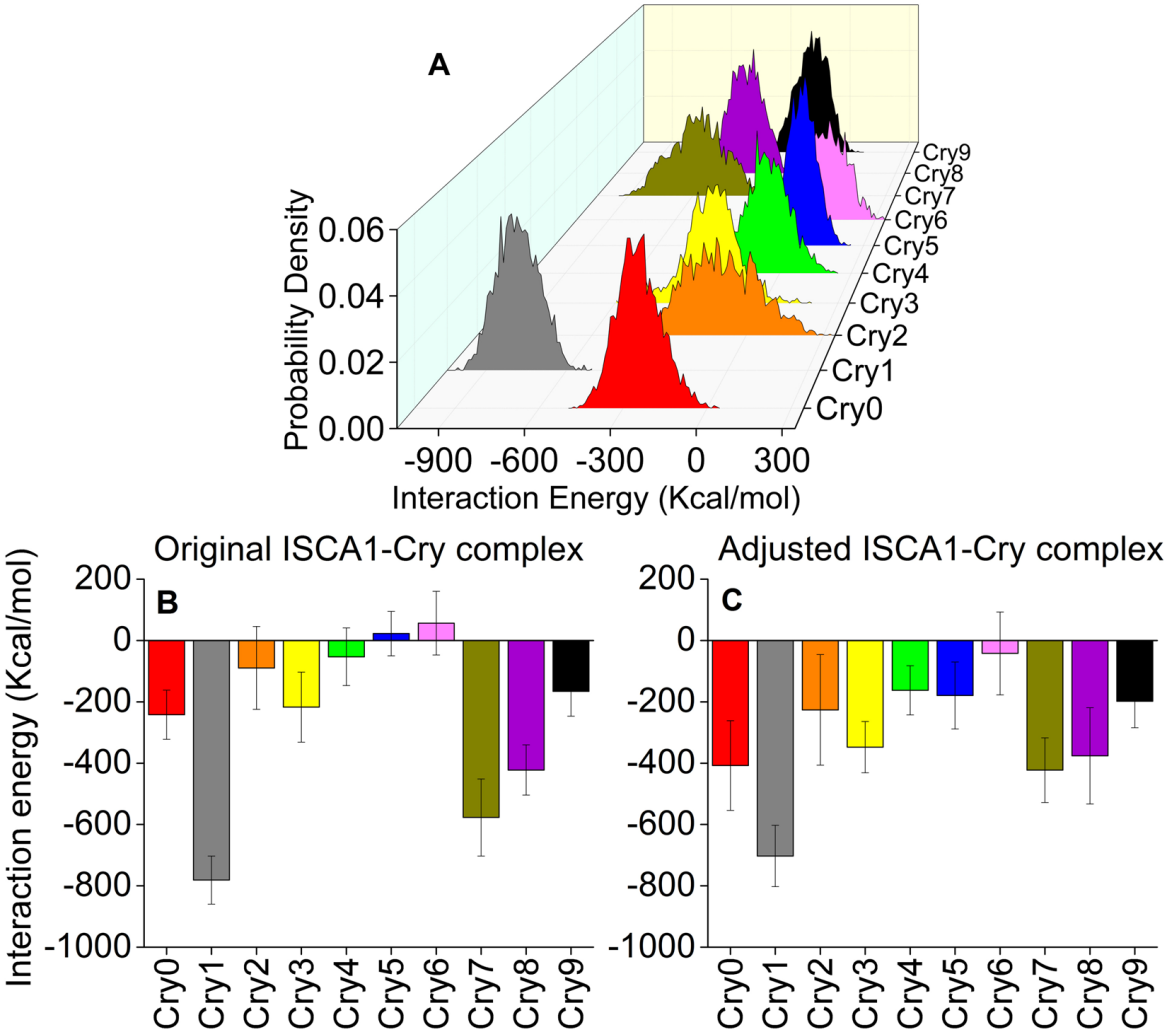
Interaction energy between cryptochromes and the ISCA1-polymer. (**A**) Shown are the distributions of the interaction energy between each individual cryptochrome and the ISCA1-complex. The distribution of interaction energies are gaussian-like which indicates local stability in the binding motif between cryptochromes and the ISCA1-polymer. The mean interaction energy shown in (**B**) for each individual cryptochrome in their original simulation, and in (**C**) for each individual cryptochrome from the simulation with adjusted configuration. Three binding modes of the cryptochromes are identified: A strong (Cry1), *E* ≤ −700 ± 100 kcal/mol, an intermediate (Cry0, Cry2, Cry3, Cry7, Cry8, Cry9), −200 ± 100 kcal/mol < *E* < −700 ± 100 kcal/mol, and a weak binding (Cry4, Cry5, Cry6), *E* ≥ −200 ± 100 kcal/mol, as well as a tendency for the outhermost cryptochromes to be stronger bound.

Cryptochrome binding affinity to the ISCA1 polymer could be characterized through the ISCA1-cryptochrome interaction energy. Figure 5A shows that the probability density distributions of interaction energy between individual cryptochromes and ISCA1 feature gaussian profiles for all cryptochromes in the system. The dynamics of the individual cryptochromes shows that most of the proteins exhibit a different interaction with the ISCA1-polymer, that could be classified into three different binding modes: a weak, a strong and an intermediate one as indicated by their mean energy, *E*, shown in Fig. 5B. The weak bound is described with *E* ≥ −200 ± 100 kcal/mol, the intermediate bound with −200 ± 100 kcal/mol < *E* < −700 ± 100 kcal/mol, and the strong bound with *E* ≤ −700 ± 100 kcal/mol. To ensure that the measured interaction energies are not an artefact of the initial starting configuration, the binding configuration of the cryptochrome with the lowest binding energy, namely Cry1 (grey), was manually imposed to the remaining cryptochromes, and the new, adjusted ISCA1-Cry complex was simulated anew. The results are compiled in Fig. 5C, which reveals that the calculated cryptochrome-ISCA1 binding energies that follow from the new simulation in fact do not change significantly as compared to the original values. It should also be noted that the behaviour of the time evolution of the RMSD and dihedral energy of the adjusted ISCA1-Cry complex, shown in Fig. 2, is not vastly different than for the original ISCA1-Cry structure.

The mean interaction energies of individual cryptochromes allow to conclude that the cryptochromes with the weakest binding are the ones located in the center of the ISCA1-polymer (Cry4, Cry5, Cry6), cryptochrome experiencing intermediate binding energy are found at the ends of the ISCA1-polymer (Cry0, Cry2, Cry3, Cry7, Cry8, Cry9), while a single cryptochrome (Cry1) distinctly stronger interaction with the ISCA1 polymer. This indicates that the geometrical shape of the ISCA1 polymer has an effect on the binding properties of the docking sites, as the ISCA1-polymer bends in a characteristic S-shape as previously discussed and shown in Fig. 3.

**Figure 6:**
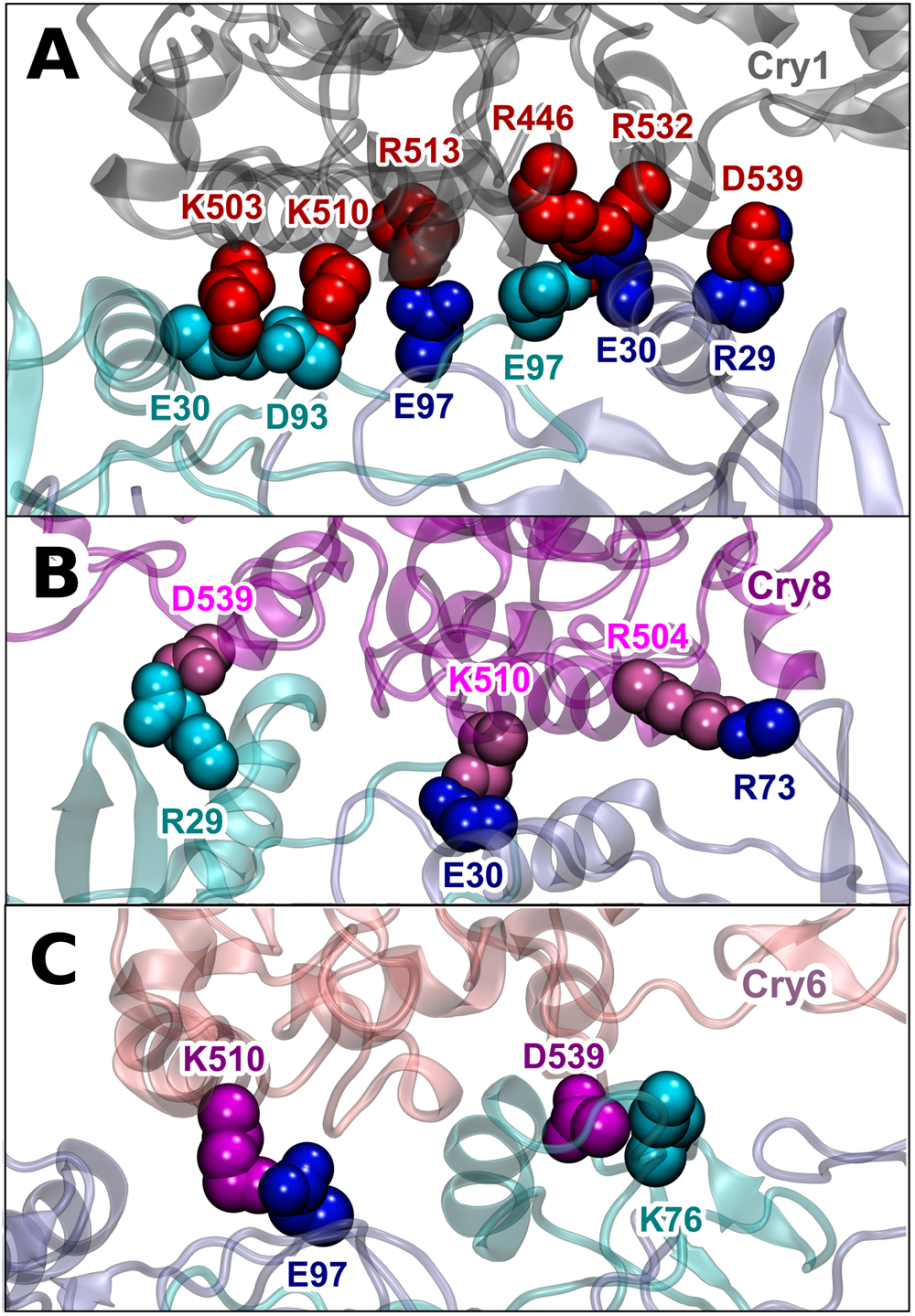
Hydrogen bondings in the strong, weak, and intermediate Cry binding modes. The specific amino acids forming hydrogen bonds are specified in the strong binding of Cry1 in (**A**) the intermediate binding of Cry8 in (**B**) and the weak binding of Cry6 in (**C**). The weaker the interaction energy, the less hydrogen bonds are formed between the Cry and the ISCA1-polymer.

The difference in binding affinity can be explained through the different hydrogen bondings formed at the interface between cryptochromes and the ISCA1 polymer. A number of different residues in the ISCA1-monomers and cryptochromes make hydrogen bonds, some turn out to be stronger than others. These hydrogen bonds characterize the binding of each cryptochrome. A weakly bound cryptochrome is characterized by having less hydrogen bonds than an intermediate or a strong binder. An example of each binding mode is shown in Fig. 6. A reoccurring hydrogen bond is D539(Cry)-R29(ISCA1) which can be found in every cryptochrome except the weakly bound Cry6.

**Figure 7:**
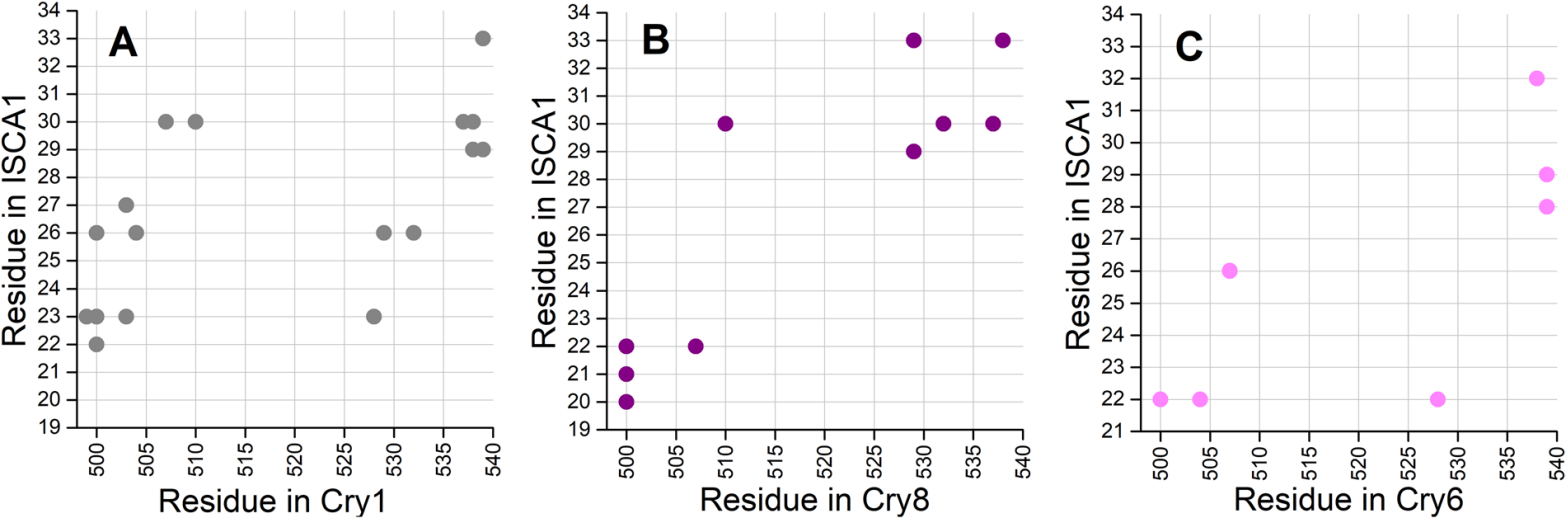
Contact maps for the strong, intermediate and weak Cry binding modes. The residues of Cry are shown on the x-axis and the residues for ISCA1 are shown on the y-asis. Dots indicates residues are within 3 Å of each other. The strong bound Cry1 is shown in (**A**) the intermediate bound Cry8 is shown in (**B**) while the weakly bound Cry6 shown in (**C**).

The different binding modes are further investigated by constructing the contact map for the interaction sites between cryptochrome and the ISCA1 polymer. The contact map indicates which amino acid residues in cryptochrome are within a distance of 3 Å of the residues of the nearest ISCA1 protein. For the purpose of the contact map the last 6 ns of simulation are analysed for the different cryptochrome binding modes using the program PyContact^46^, and the results are shown in Fig. 7. The contact map confirms the classification of binding modes as the strong bound Cry1 has more contacts than the intermediate bound Cry8, which again has more contacts than the weakly bound Cry6.

## Discussion

As the claims made by Qin *et al*.^35^ were received with scepticism^36–39^ within the field of magnetoreception, an independent study addressing the published results was called for. The present paper investigates and expands on the computational part of the Qin *et al*. study to investigate the dynamics of the proposed ISCA1-Cry complex to characterize the interaction of cryptochrome and ISCA1.

The protein polymer consisting of 24 ISCA1 monomers was assembled carefully following the procedure described by Qin *et al*.^35^. In the course of this modelling, it was discovered that ISCA1 monomers are best constructed by using a different template namely protein 2D2A^47^ instead of the proposed 1R94^48^. The two structures are highly similar, but the used 2D2A supports iron sulphur clusters whereas 1R94 is a mercury derivative.

After a careful equilibration of the ISCA1-complex, ten cryptochromes from *Drosophila melanogaster* were added to the system, forming the ISCA1-Cry complex, and the stability of this assembly was studied dynamically to obtain sufficient statistics to conclude about the interaction energy arising between cryptochromes and the ISCA1 polymer. The computations revealed that one cryptochrome was significantly stronger bound than the other nine cryptochromes, but changing their binding configuration to match that of the best binder did not change the interaction energy drastically.

The discovery of different binding modes of cryptochrome depending on its placement along the ISCA1 rod, as well as the observed high flexibility of the isolated ISCA1 and ISCA1-Cry complexes indicate that both systems are likely not robust enough to exist in a real cellular environment, at least not in the proposed form. Moreover, the extraordinarily large edge-to-edge distance of ~30–50 Å between the active site of cryptochrome (FAD and the tryptophan triad) and the nearest iron sulphur cluster from the ISCA1 polymer makes the polymer rather useless for a possible electron transfer to/from the cryptochrome and thus also unlikely to influence cryptochrome magnetoreceptive properties.

It is, however, important that the present study has computationally established the possibility for a bonding between ISCA1 and cryptochrome, as no crystallized interaction partners of cryptochrome, except those associated with its circadian rhythm function, have been reported^17,49–51^. The role of ISCA1 as an interaction partner should therefore be investigated in other systems^49,52,53^, preferably in species known to be magnetosensitive, to see if it could still facilitate electron transfer reactions in some of them.

## Methods

In this section we describe the protocol for construction of the ISCA1-polymer, and provide details on how this approach differs from the one described in the original paper^35^. We then discuss the construction of the ISCA-Cry complex and explain how cryptochromes were docked to the ISCA1 polymer surface. Finally the details of the computational methods employed in the simulations of the ISCA1 polymer and the ISCA1-Cry complex are provided.

### Molecular construction of ISCA1 and ISCA1 complexes

#### Homology modelling of ISCA1

The protein that supposedly aggregates into a rod-like structure and binds to cryptochrome^35^, is found in *Drosophila melanogaster* and is an iron sulfur containing protein, CG8918^54^, with an unknown function. The structure of CG8918 from *Drosophila melanogaster* has not been experimentally resolved, therefore a homology model of the protein was build, using the phyre2 server^55^. The best template for the homology model was found to be *Escherichia coli* SufA (PDB-ID: 2D2A^47^), involved in biosynthesis of iron-sulfur clusters, with an amino acid sequence identity with CG8918 being 95%.

**Figure 8:**
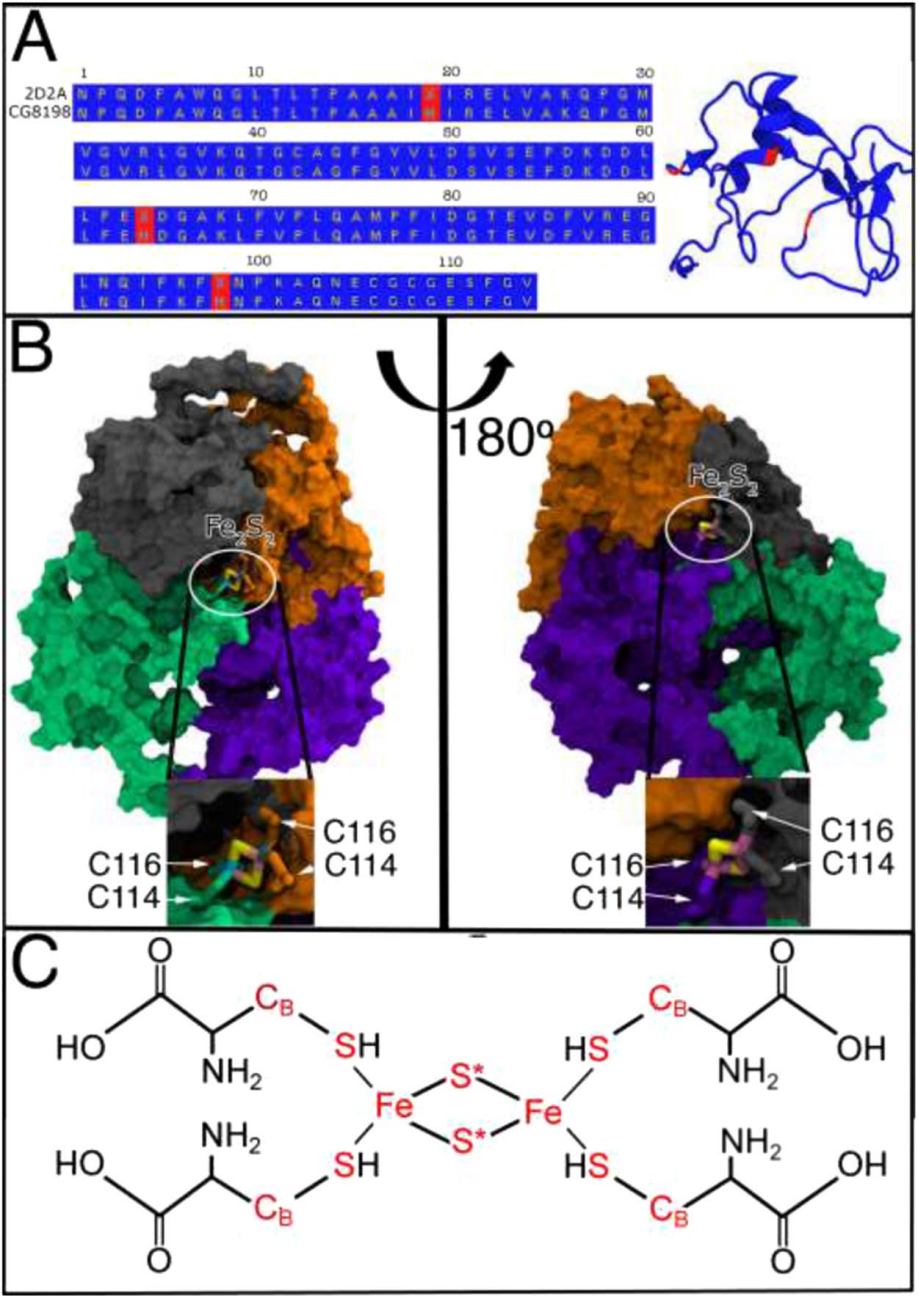
Constructing ISCA1. (**A**) Amino acid alignment of CG8918 and 2D2A protein. 2D2A^47^ was used as a template for modelling the CG8918 monomer, due to the 95% sequence similarity. Red labels indicate different amino acids while blue labels show similar ones. The resulting homology model of CG8918 is shown in the secondary structure representation, coloured according to the amino acid similarity with the template. (**B**) Graphical depiction of the interconection between the CG8918 monomers, facilitated by the iron sulfur (Fe_2_S_2_) clusters. Four monomers, shown as surfaces in green, purple, orange and grey, are cross-linked by two Fe_2_S_2_-clusters, which are coordinated by four cysteins, residues 116 and 114. (**C**) Chemical formula of the four coordinating cysteins and the iron sulphur cluster. Atoms marked red indicates those for which the partial charges are provided in Table 1.

The original study by Qin *et al*. proposed an iron-sulfur cluster assembly protein from *Escherichia coli* (PDB-ID: 1R94^48^) as the homology model template, however, this template for the ISCA1 monomers was not found among the top 50 suggested templates on the phyre2 server. It is worth noting that the crystal structure of 1R94 does not contain iron-sulfur clusters, but is a mercury derivative, whereas 2D2A features clear indications as to where Fe_2_S_2_, iron-sulfur clusters, should be positioned. Figure 8A, left, shows the amino acid allignment of CG8198 and the template 2D2A, where red and blue indicate different and identical amino acid residues, respectively. The resulting homology model can be seen in the secondary structure representation in Fig. 8A, right, coloured according to the amino acid identity with the template, as in the case of the amino acid sequence alignment. The high degree of similarity indicates that the structure of the CG8198 protein is likely highly similar to the chosen template. Furthermore, two of the three different amino acid residues are located on random coil motifs, while the third is located at the beginning of an alpha helix, which will likely not have a big effect on the secondary structure of the entire protein.

#### Building the ISCA1-polymer

The CG8198 monomers were proposed to aggregate into a rod-like polymer structure, based on the crystal packing structure of the 1R94 protein^35^. The crystal structure of the 2D2A protein does not have the same crystal packing and, therefore, to be able to test the stability of the structure proposed earlier^35^, we have used the crystal packing structure from 1R94 instead. A 2 × 2 × 2 unit cell of the crystal packing of the 1R94 protein was created using the Chimera software package^56^ and a polymer containing 24 monomers was extracted. Each 1R94 monomer was then replaced with the homology model of CG8198 derived from the 2D2A structure, yielding the ISCA1-polymer rod structure. The VMD software package^57^ was subsequently used for further 2D2A structure analysis.

Upon inspection of the structure, two cysteines, C116 and C114, were found as the only candidates to coordinate the iron-sulfur clusters, as seen in Fig. 8C. The figure shows how the monomers of the ISCA1 polymer are cross-linked by iron-sulfur clusters, coordinated by two cysteines from each monomer; Fig. 8B shows monomers as surfaces of different colour, while Fig. 8C shows a schematics of the iron-sulphur cluster-cryptochrome binding.

#### Cryptochrome docking

The final step in constructing the ISCA1-Cry complex is the docking of cryptochrome proteins to the ISCA1-polymer. In this study we have used cryptochrome from *Drosophila melanogaster* (PDB-ID: 4GU5^58^), which has been structurally equilibrated previously^59^. The docking of cryptochrome to the ISCA1 is accomplished following the protocol prepared by Qin *et al*.^35^, based on the crystal packing structure of the 1R94 protein, where the 1R94 monomers are non-covalently bound to cryptochromes through four alpha helices. Two similar alpha helices are found in *Drosophila melanogaster* cryptochrome, which are then placed according to the two helices in the crystal packing structure of the ISCA1 polymer. In total 10 cryptochrome proteins were docked to the ISCA1-polymer, and the resulting structure can be seen in Fig. 1A, where the cryptochrome proteins are shown with surfaces, labeled as Cry0-Cry9, and the ISCA1-polymer is shown in pale blue.

### Molecular dynamics simulations

The stability and dynamics of the ISCA1 and ISCA1-Cry structures were investigated through MD simulations, performed employing the program NAMD 2.11^42^. The CHARMM36 force field for proteins with CMAP corrections^60^ was used in the simulations. The parameters for FAD in the cryptochrome were developed by us in a series of earlier studies^31,43,61,62^, while the interaction parameters for iron sulphur clusters were taken from the literature^63–65^. An additional set of charges were obtained by doing quantum mechanical calculations of the electrostatic potential of the optimized iron sulphur cluster coordinated by four cystein amino acids by employing the software Gaussian^66^ and chemical symmetry considerations. The obtained set of charges appear to be similar to the ones deprived earlier which justifies their use for all the performed simulations. For the sake of completeness both sets of partial charges of the atoms from the iron sulphur cluster its coordinating cysteins are compiled in Table 1.

**Table 1:** Partial charges for the atoms in the iron sulphur cluster and coordinating cysteins.

Atom	S*	Fe	S	C_*B*_
Mouesca *et al*.^63^	−0.61	0.76	−0.59	−0.16
ESP fitted charges	−0.69	0.78	−0.68	−0.04

S* and Fe denote the atoms of the iron sulphur cluster whereas S and C_*B*_ are the sulphur and the beta-carbon atom of the coordinating cystein; a schematic of the Fe_2_S_2_-cystein complex can be found in Fig. 1C. The hydrogens in the cysteins are assigned partial charges of 0.09 according to the CHARMM force field nomenclature^59^.

Periodic boundary conditions were used in all MD simulations and the particle-mesh Ewald (PME) summation method was employed for evaluating Coulomb interactions^67^. The van der Waals energy was calculated using a cutoff distance of 12 Å. Analysis of the simulations was carried out using the program VMD 1.9.2^57^. The simulations were preformed assuming the temperature of 300 K by utilizing the Langevin temperature control with a damping coefficient of 5 ps^−1^ as well as Nosé-Hover-Langevin piston pressure control^68^ with a period of 200 fs and a decay of 50 fs, keeping the pressure at 1 atm.

A complete summary of the equilibration protocol of the performed simulations is compiled in Table 2. All MD simulations were preceded by a structure optimization taking 10,000 NAMD steps. More specific details on these simulations are provided below.

**Table 2:** The equilibration process for the ISCA1 polymer and the ISCA1-Cry complex.

Structure	Constraint	Subsystem to be equilibrated	Simulation time (ns)
ISCA1 polymer	ISCA1 structure	Water	0.1
	Not random coils	Random coils	20
	Backbone	Sidechains	7
	None	ISCA1-structure	50
	None	Production run	150
Original ISCA1-Cry complex	ISCA1-Cry structure	Water	0.01
	None	Entire ISCA1-Cry structure	50
	None	Production run	150
Adjusted ISCA1-Cry complex	ISCA1-Cry structure	Water	0.01
	None	Entire ISCA1-Cry structure	50
	None	Production run	150

### ISCA1-polymer simulations

The assembled ISCA1 polymer was proposed as a possible binding partner to cryptochrome^35^. However, since the ISCA1 polymer is not a known structure, its construction is likely far from a stable configuration. Thus it needs to be equilibrated before it is made to interact with the cryptochromes. This is a crucial point in validating the possible ISCA1 polymer-cryptochrome binding which was not performed in the original paper^35^.

To prepare the system for production run, the ISCA1 polymer was placed in a water box with a salt concentration of 0.15 mol/L NaCl yielding a total atom count of 573,789. After a minimization process of 10,000 NAMD steps, constraints were put on the system and gradually released to make sure the ISCA1-polymer would equilibrate, see Table 2. The first equilibration stage allowed the water molecules and ions to settle while the ISCA1-polymer was constrained during a time period of 0.1 ns. Afterwards the flexible random coils were released for 20 ns to allow them to equilibrate. The next stage assured the side chains of the protein to be relaxed, only leaving constraints on the polymer’s backbone atoms during a simulation process of 7 ns. Finally all constrains on the entire ISCA1 polymer were released, making it stable enough to increase the time-step from 1 fs to 1.5 fs, where it was subject to another equilibration for another 50 ns before the production simulation was started. A summary of the equilibration process can be found in Table 2.

### ISCA1-Cry complex simulations

After the ISCA1-polymer was simulated in production for ~10 ns, cryptrochromes were added to the surface forming the composite ISCA1-Cry structure. The ISCA1-Cry structure was then resolvated in a new waterbox with a salt concentration of 0.15 mol/L NaCl, yielding a total atom count of 1,957,756. Since the ISCA1-polymer, which is the central part of the ISCA1-Cry-structure, was carefully equilibrated before adding the pre-equilibrated cryptochromes from an earlier investigation, the equilibration protocal was reduced to now including 10,000 NAMD minimization steps and a short but sufficient equilibration of the water around the ISCA1-Cry structure. The entire protein complex was then released and allowed to equilibrate for further 50 ns prior production simulations.

After a 150 ns long production simulation, the cryptochromes were found to bind to the ISCA1-polymer with different strengths, see results below. To check if this is an artefact of the simulation or an actual biological behaviour, the position after 100 ns of the cryptochrome with the lowest binding energy, (Cry1), was assumed for the other nine cryptochromes and the simulation was repeated for the adjusted configuration for 150 ns after a 50 ns equilibration period.
